# Real-World Comparative Effectiveness of First-Line Dabrafenib Plus Trametinib Versus Immunochemotherapy in Patients with Advanced BRAF V600-Mutant Non-Small-Cell Lung Cancer: A Retrospective Cohort Study

**DOI:** 10.3390/jcm15176749

**Published:** 2026-08-31

**Authors:** Haizhou Yue, Qianxin Zhou, Suyu Wang, Shuangyi Li, Junyi Li, Huanzhi La, Shuyan Meng

**Affiliations:** 1 Department of Thoracic Surgery, Shanghai Pulmonary Hospital, School of Medicine, Tongji University, Shanghai 200433, China; yuehaizhou87@163.com (H.Y.); zhouqxin01@163.com (Q.Z.); wangsuyv357311854@163.com (S.W.); 2011353@tongji.edu.cn (S.L.); junyi_li@tongji.edu.cn (J.L.); 2111830@tongji.edu.cn (H.L.); 2 Department of Medical Oncology, Shanghai Pulmonary Hospital, School of Medicine, Tongji University, Shanghai 200433, China

**Keywords:** BRAF V600 mutation, NSCLC, targeted therapy, real-world evidence

## Abstract

**Background**: The optimal first-line treatment for advanced BRAF V600-mutant non-small cell lung cancer (NSCLC) remains uncertain. We compared the real-world effectiveness of first-line Dabrafenib plus trametinib (Dab+Tram) with immunochemotherapy (ICT) and other systemic regimens. **Methods**: This single-centre retrospective cohort study included 142 patients with unresectable stage III/IV BRAF-mutant NSCLC treated between April 2017 and May 2024. Patients were grouped by first-line regimen Dab+Tram, ICT, chemotherapy alone, or others. Primary endpoint was real-world progression-free survival (rwPFS). Propensity score matching (PSM) and multivariable Cox regression were applied to reduce measured confounding. **Results**: Forty-seven patients received Dab+Tram and 30 received ICT. In unadjusted analysis, Dab+Tram yielded significantly longer median rwPFS than ICT (32.5 vs. 13.3 months; *p* = 0.004). After PSM (25 pairs), median rwPFS was 19.6 months for Dab+Tram versus 12.6 months for ICT (HR = 1.74, 95%CI 0.85–3.66, *p* = 0.12). Multivariable Cox regression showed ICT, chemotherapy alone, and other regimens were independently associated with worse rwPFS versus Dab+Tram (all *p* < 0.05). Overall survival showed no significant difference. Dab+Tram showed some antitumor activity in later-line settings. Findings regarding pyrexia and PD-L1 subgroups were exploratory. **Conclusions**: In this real-world cohort, first-line Dab+Tram suggested a potential rwPFS benefit over ICT and chemotherapy. The matched comparison did not reach statistical significance, but multivariable analysis consistently supported an association between Dab+Tram and improved rwPFS. These findings provide complementary real-world evidence and require prospective validation.

## 1. Introduction

Lung cancer remains a leading cause of cancer-related mortality worldwide [1]. NSCLC accounts for approximately 80–85% of all lung cancer cases, and more than 60% of patients present with advanced-stage disease at initial diagnosis [2,3]. BRAF V600 mutations define a rare but clinically actionable molecular subgroup of NSCLC [4]. Dab+Tram, a combined BRAF and MEK inhibition strategy, has demonstrated substantial antitumour activity in patients with advanced BRAF V600-mutant NSCLC and is an established targeted treatment option for this population [5]. However, in routine clinical practice, the optimal first-line treatment strategy remains uncertain, particularly because immunochemotherapy (ICT) is also widely used for advanced NSCLC and may provide durable benefit in selected patients [6].

Previous real-world studies have compared BRAF/MEK inhibition with broadly defined immunotherapy-based strategies in patients with BRAF V600-mutant NSCLC [7]. However, these comparator groups frequently included both immune checkpoint inhibitor monotherapy and ICT, introducing substantial treatment heterogeneity. Evidence specifically comparing first-line Dab+Tram with a homogeneous ICT strategy remains limited, particularly in Chinese patients. This distinction is clinically relevant because ICT represents a commonly used competing first-line option in routine practice [8].

Pyrexia is a common treatment-emergent adverse event during Dab+Tram therapy [5]. Whether treatment-emergent pyrexia is associated with clinical outcomes in patients with BRAF V600-mutant NSCLC remains unclear. Given the limited available clinical evidence, its potential prognostic relevance was explored in the present study.

Therefore, we conducted a single-centre retrospective real-world cohort study to compare the effectiveness of first-line Dab+Tram with ICT in patients with advanced BRAF V600-mutant NSCLC. PSM and multivariable Cox regression were applied to address measured confounding. We also descriptively evaluated the activity of Dab+Tram in subsequent-line settings and explored the potential association between treatment-emergent pyrexia and clinical outcomes.

## 2. Materials and Methods

### 2.1. Study Design and Patient Population

This was a single-centre, retrospective observational study conducted at Shanghai Pulmonary Hospital, School of Medicine, Tongji University. We consecutively enrolled adult patients (≥18 years) with histologically confirmed, unresectable locally advanced (stage III) or metastatic (stage IV) NSCLC who were treated between April 2017 and May 2024. All patients had a confirmed BRAF mutation detected by NGS or PCR.

Inclusion criteria: age ≥18 years; histologically or cytologically confirmed NSCLC; presence of a BRAF mutation confirmed by next-generation sequencing (NGS) or polymerase chain reaction (PCR); clinical stage unresectable locally advanced (stage III) or IV disease; treated at our institution between April 2017 and May 2024; and availability of complete treatment and follow-up records.

Exclusion criteria: absence of BRAF mutation; lack of essential treatment or survival data; loss to follow-up immediately after treatment initiation.

### 2.2. Ethics Approval and Informed Consent

This retrospective study was approved by the Ethics Committee of Shanghai Pulmonary Hospital (Approval No. K25-538), with a waiver of written informed consent given the retrospective nature of the study, and all procedures were performed in accordance with the Declaration of Helsinki.

### 2.3. Data Collection and Variable Definitions

Clinical data were systematically extracted from the electronic medical record system, including age, gender, smoking history, body mass index, Eastern Cooperative Oncology Group performance status, disease stage, histological subtype, metastatic sites, PD-L1 tumour proportion score when available, genomic alterations, treatment regimens, treatment lines, subsequent therapies, progression status, and survival outcomes.

First-line treatment groups were defined according to the initial systemic regimen received for advanced disease: dabrafenib plus trametinib; immunochemotherapy (ICT), defined as a PD-1 or PD-L1 inhibitor administered in combination with platinum-based chemotherapy; chemotherapy alone; and other systemic regimens. Patients receiving immune checkpoint inhibitor monotherapy were not included in the ICT comparator group.

### 2.4. Outcome Measures

The primary endpoint was rwPFS, defined as the time from initiation of first-line systemic treatment to investigator-documented disease progression or death from any cause, whichever occurred first. Patients without progression or death were censored at the date of their last available disease assessment. Secondary endpoints included overall survival (OS), defined as the time from initiation of first-line treatment to death from any cause, and rwPFS among patients receiving dabrafenib plus trametinib in subsequent-line settings. Exploratory endpoints included outcomes according to treatment-emergent pyrexia and PD-L1 expression status.

### 2.5. Statistical Analysis

Baseline characteristics were compared using *t*-test/Wilcoxon rank-sum and chi-square/Fisher’s exact tests. rwPFS and OS were estimated by the Kaplan–Meier method with the log-rank test; Cox models estimated HRs and 95% CIs (proportional hazards assumption checked by Schoenfeld residuals). To reduce confounding, propensity score matching (1:1, caliper = 0.2) was performed using logistic regression with age, gender, ECOG status, and disease stage; balance was assessed by standardised mean differences (<0.10). Multivariable Cox regression further adjusted for baseline prognostic factors. Given the observational design, HRs were interpreted as associations, not causal effects. Analyses according to PD-L1 expression, treatment-emergent pyrexia, and subsequent-line use of dabrafenib plus trametinib were prespecified as exploratory. For pyrexia analyses, survival outcomes were calculated from baseline. Although a 4-week landmark analysis was initially planned to reduce guarantee-time bias, it was not performed because all pyrexia events occurred within the first 4 weeks of treatment and no patients were excluded at the landmark. All statistical tests were two-sided, and *p* < 0.05 was considered statistically significant (R version 4.2.0).

## 3. Results

### 3.1. Baseline Characteristics

A total of 142 patients were included in the analysis. The median age was 65.0 years (IQR: 58.0–71.0), and 60.6% (86/142) were male. The vast majority of patients (97.2%, 138/142) had adenocarcinoma histology, and 86.6% (123/142) presented with stage IV disease at initial diagnosis. Extrathoracic metastases were present in 36.6% (52/142) of patients, with the pleura, bone, brain, and adrenal glands being the most common sites (Table 1).

**Figure 1:**
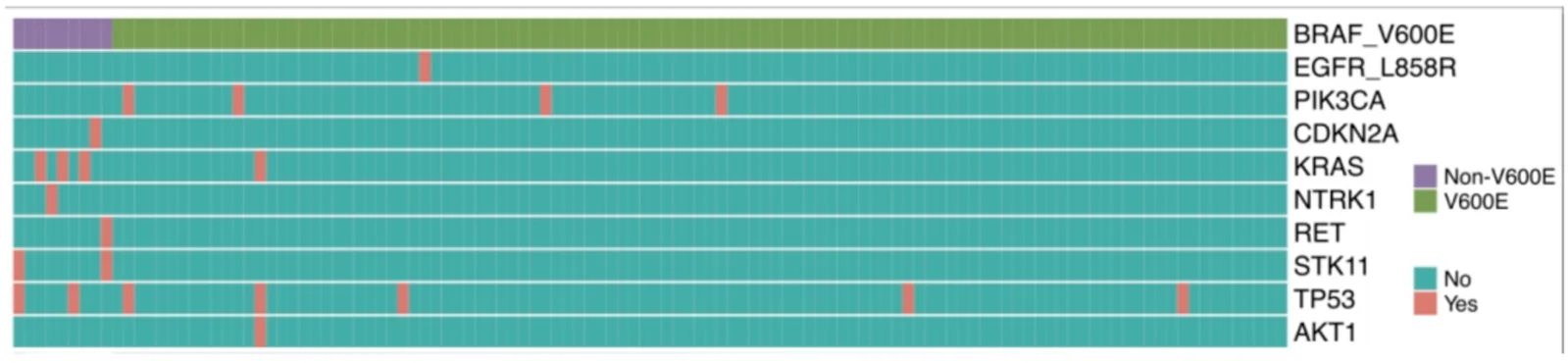
Baseline concomitant mutations with BRAF mutation detected by PCR or NGS.

A total of 142 patients were included in the analysis. BRAF mutation status was available for 116 patients (81.7%) and was the basis for all subsequent mutation-related analyses. Among patients with known BRAF mutation status (*n* = 116), V600 mutations were predominant (94.0%, 109/116), with the V600E subtype accounting for the majority. Among the 7 patients with non-V600E mutations, 2 received ICT, 3 received chemotherapy alone, and 2 received other immune-based chemotherapy-free regimens. Concomitant mutations were detected in 12.9% (15/116) of patients, with TP53 being the most common (6.0%) (Figure 1). Baseline characteristics were generally well-balanced across the four first-line treatment cohorts (Table 1).

### 3.2. Treatment Patterns and Primary Endpoint (rwPFS)

**Figure 2:**
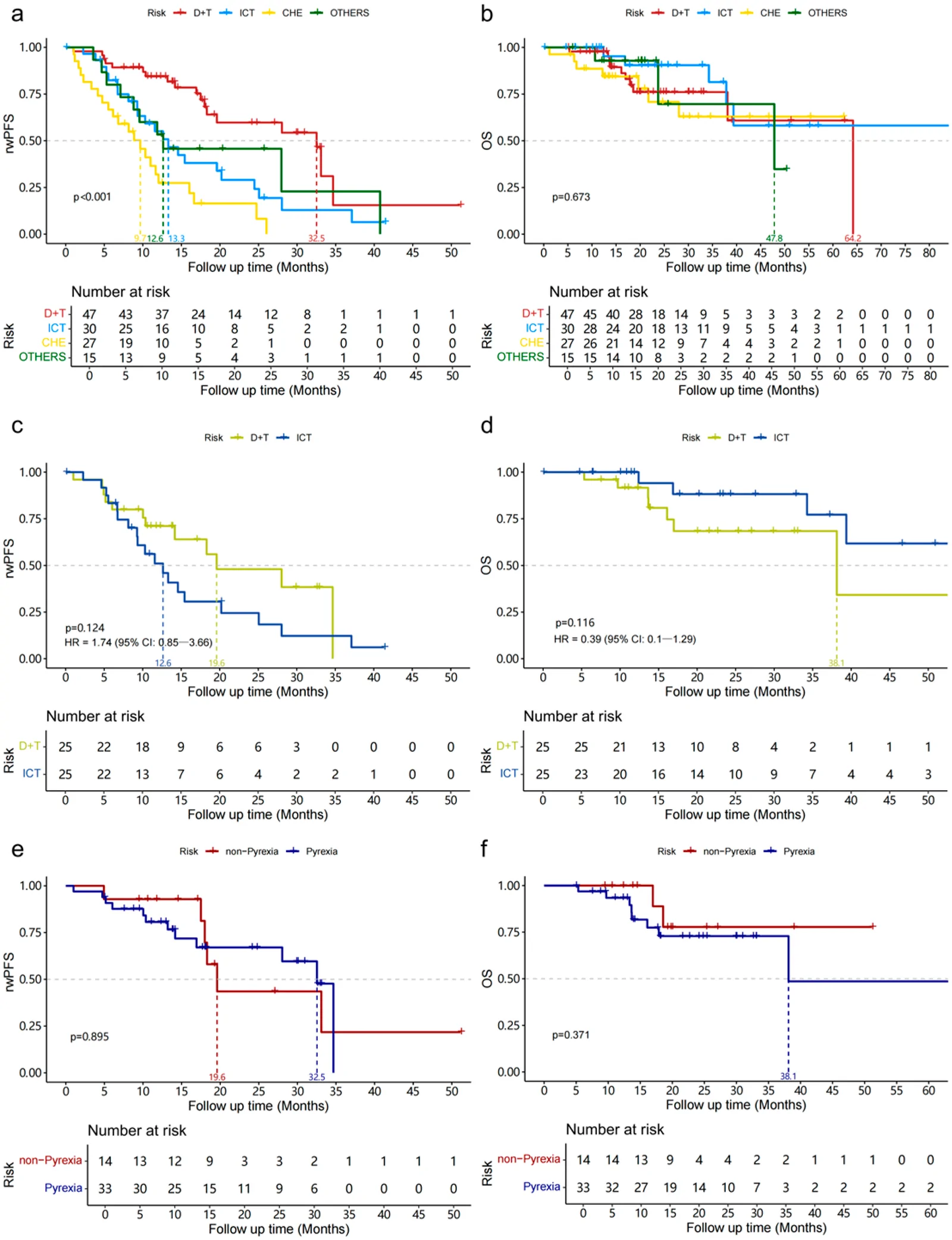
rwPFS and OS in patients with BRAF V600-mutant NSCLC. rwPFS for first-line Dab+Tram, ICT, chemotherapy, or other regimens with risk table (**a**,**b**); rwPFS (**c**) and OS (**d**) after PSM for Dab+Tram versus ICT; rwPFS (**e**) and OS (**f**) by treatment-emergent pyrexia in the Dab+Tram cohort.

Dab+Tram (33.1%, 47/142) and ICT (21.1%, 30/142) were the most frequently used first-line regimens. First-line Dab+Tram demonstrated significantly longer median rwPFS compared with chemotherapy alone (32.5 months [95% CI: 18.3–NR] vs. 9.7 months [95% CI: 6.1–16.0]; *p* < 0.001) and ICT (13.3 months [95% CI: 9.3–24.5]; *p* = 0.004) (Figure 2a). Among patients with locally advanced disease (stage III, n = 13) who received first-line therapy, Dab+Tram achieved a median rwPFS of 34.7 months (95% CI: 28.9–NR), which was significantly longer than the 3.4 months (95% CI: 1.2–6.8) observed with chemotherapy (*p* < 0.001) (Supplementary Figure S1).

### 3.3. Survival Analysis, PSM

After a median follow-up of 22.6 months, the survival differences between the first-line Dab+Tram group and the ICT or chemotherapy groups did not reach statistical significance, partly due to crossover to Dab+Tram upon disease progression in some patients from the latter groups (Figure 2b). To adjust for potential confounding bias, we performed PSM. Given the limited sample size, covariates included age, gender, ECOG performance status, and clinical stage. After matching, 25 patients were included in each group, and baseline characteristics were well balanced (Table 2). In the matched cohort, the median follow-up duration was 23.3 months. The median rwPFS of the ICT group was shorter than that of the Dab+Tram group (12.6 months vs. 19.6 months; HR = 1.74, 95% CI: 0.85–3.66, *p* = 0.12) (Figure 2c), and the difference did not reach statistical significance. The median overall survival (OS) also showed no statistically significant difference between the two groups (HR = 0.39, 95% CI: 0.10–1.29, *p* = 0.12) (Figure 2d).

### 3.4. Prognostic Risk Factors of Survival

In univariate Cox regression analysis, ECOG performance status (HR = 4.872, 95% CI: 1.279–13.690, *p* = 0.024) and extrathoracic metastasis (HR = 2.665, 95% CI: 1.227–5.785, *p* = 0.014) were significantly associated with worse OS, while ICT (HR = 2.533, 95% CI: 1.363–4.764, *p* = 0.003) and chemotherapy alone (HR = 5.074, 95% CI: 2.684–9.734, *p* < 0.001) were significantly associated with worse rwPFS compared with Dab+Tram.

Multivariate Cox regression analysis (Table 3) showed that, compared with first-line Dab+Tram treatment, ICT (HR = 2.500, 95% CI: 1.322–4.780, *p* = 0.005), chemotherapy alone (HR = 5.534, 95% CI: 2.90–10.706, *p* < 0.001), and other regimens (HR = 2.652, 95% CI: 1.170–5.724, *p* = 0.021) were independently associated with significantly worse rwPFS. In addition, extrathoracic metastasis (HR = 2.029, 95% CI: 1.152–3.540, *p* = 0.015) was also an independent unfavourable prognostic factor for rwPFS. Regarding overall survival (OS), no variable reached statistical significance in the multivariate model (all *p* > 0.05). This finding may be largely attributable to crossover to Dab+Tram therapy after disease progression in some patients originally treated with ICT or chemotherapy, which confounded the between-group differences in OS. Furthermore, the limited sample size may have also reduced statistical power to some extent.

### 3.5. Exploratory Analysis: Treatment-Emergent Pyrexia and Efficacy

All 47 patients who received first-line Dab+Tram were included in the pyrexia subgroup analysis, with no patients excluded. The median rwPFS and OS values were calculated from baseline. Among the 47 patients treated with first-line Dab+Tram, 33 (70.2%) experienced treatment-emergent pyrexia. Although the difference did not reach statistical significance (*p* = 0.895), a trend toward longer median rwPFS was observed in patients with pyrexia compared with those without (32.5 months [95% CI: 19.8–NR] vs. 19.6 months [95% CI: 10.2–28.9]) (Figure 2e). No significant difference in OS was observed between the two groups (median OS: 42.3 months vs. 38.7 months; *p* = 0.371) (Figure 2f).

### 3.6. Exploratory Subgroup Analysis by PD-L1 Expression

In the PD-L1 1–49% subgroup, Dab+Tram showed significantly longer rwPFS compared with ICT (*p* = 0.034). However, this finding is based on extremely small sample sizes (e.g., *n* = 7 vs. 7 in the 1–49% subset) and should be interpreted with caution, as it is highly susceptible to small-sample bias. No consistent treatment difference was observed across PD-L1 categories, and these results should not be used to guide clinical decision-making (Supplementary Figure S2).

### 3.7. Efficacy of Subsequent-Line Therapies

Dab+Tram continued to show meaningful activity in later-line settings, with a median rwPFS of 15.1 months in 14 patients receiving second-line therapy and 7.6 months in 9 patients receiving third-line or beyond. Among the 30 patients initially treated with ICT, 8 (26.7%) subsequently received Dab+Tram as second-line or later therapy upon disease progression. Among the 27 patients initially treated with chemotherapy alone, 6 (22.2%) subsequently received Dab+Tram. This rate of crossover to BRAF/MEK inhibition may have confounded the OS comparison between treatment groups. These findings suggest a potential clinical benefit of Dab+Tram as subsequent therapy after first-line non-targeted treatment failure in BRAF V600-mutant NSCLC (Supplementary Figure S3).

## 4. Discussion

This single-centre retrospective real-world analysis evaluated the efficacy of first-line Dab+Tram in patients with advanced BRAF V600-mutant NSCLC. In the unadjusted analysis, the Dab+Tram group achieved a median rwPFS of 32.5 months, significantly longer than that of chemotherapy (9.7 months) and ICT (13.3 months). However, selection bias inherent to retrospective studies may inflate treatment effects. To address this, we performed PSM including age, gender, ECOG performance status and clinical stage as covariates, successfully matching 25 patient pairs with well-balanced baseline characteristics (Table 2). In the matched cohort (median follow-up 23.3 months), median rwPFS was 19.6 months for Dab+Tram and 12.6 months for ICT (HR = 1.74, 95%CI: 0.85–3.66, *p* = 0.12). Although not statistically significant, the effect size (HR = 1.74) is clinically meaningful. OS also showed no statistically significant difference (HR = 0.39, 95%CI: 0.10–1.29, *p* = 0.12).

The key innovation of this study lies in the choice of comparator: whereas previous real-world studies have largely compared BRAF/MEK inhibitors with broadly defined immunotherapy cohorts [7,9] (including ICI monotherapy with or without chemotherapy), we specifically selected ICT as the comparator, thereby focusing on a more clinically homogeneous and relevant question concerning first-line treatment selection. Our findings are consistent with other real-world evidence-the French REBRAFR study (median PFS 16.8 months) [10], a U.S. electronic health record analysis (≈20 months) [11] and a Chinese multicentre retrospective study (25.0 months) [7] all support the first-line value of Dab+Tram. Our PSM-adjusted median rwPFS of 19.6 months aligns closely with these data, indicating that the efficacy of Dab+Tram is robust with little variation across populations. To further assess potential unmeasured confounding, we calculated the E-value. An E-value analysis for the PSM-adjusted HR of 1.74 yielded an E-value of 2.87, suggesting that fairly strong unmeasured confounding would be required to explain the observed effect. Nevertheless, given the wide confidence interval and the observational design, residual confounding cannot be ruled out.

Multivariable Cox regression analysis (Table 3) showed that, compared with first-line Dab+Tram, ICT (HR = 2.50, *p* = 0.005), chemotherapy alone (HR = 5.534, *p* < 0.001), and other regimens (HR = 2.652, *p* = 0.021) were independently associated with significantly worse rwPFS; extrathoracic metastasis (HR = 2.029, *p* = 0.015) were also independent unfavourable prognostic factors. No variable reached statistical significance for OS, possibly owing to subsequent crossover to targeted therapy and the limited sample size after PSM (25 pairs). After PSM, the rwPFS difference was not statistically significant (HR = 1.74, *p* = 0.12) and should be interpreted as a numerical trend due to limited sample size. Nevertheless, multivariable Cox analysis independently supported an association between Dab+Tram and improved rwPFS (HR = 2.50, *p* = 0.005). Overall, these findings suggest a numerical trend favouring Dab+Tram that warrants prospective validation.

Our findings should be interpreted in the context of the recently published FRONT-BRAF study [9]. FRONT-BRAF demonstrated that, in patients with BRAF V600E-mutant advanced NSCLC, first-line immune checkpoint inhibitor therapy (with or without chemotherapy) significantly prolonged OS compared with Dab+Tram (40.9 vs. 25.2 months; HR = 0.69, *p* = 0.039), whereas rwPFS was similar between the two strategies. In contrast, our unadjusted analysis showed longer rwPFS with first-line Dab+Tram than with ICT, whereas this difference was attenuated and no longer statistically significant after propensity score matching. No statistically significant OS difference was observed. Several differences in study design, comparator composition, and patient characteristics may help explain the different patterns of treatment outcomes across studies. First, the immunotherapy arm in FRONT-BRAF included both ICI monotherapy (61%) and ICT, whereas our comparator was restricted to ICT alone; this difference in treatment composition may attenuate the rwPFS advantage of targeted therapy over immunotherapy. Second, the FRONT-BRAF population had higher proportions of ever-smokers and PD-L1 ≥50% expression, both associated with greater benefit from immunotherapy [12,13]. Smoking status is a well-established effect modifier of immunotherapy efficacy, with smokers deriving greater benefit from immune checkpoint inhibitors, likely due to higher tumour mutational burden and neoantigen load. In our study, smoking history could not be incorporated into the PSM model. Consequently, we could not ascertain whether baseline smoking distribution was balanced between treatment groups, nor to what extent the observed ICT efficacy reflected the immunological advantage conferred by smoking status rather than the direct effect of the ICT regimen itself. This limitation is particularly relevant when contextualising our findings alongside the FRONT-BRAF trial, where 83% of patients were ever-smokers. The differential smoking profiles across studies may partly account for the divergent outcomes observed, beyond the treatment strategies per se. Accordingly, our interpretation of ICT efficacy warrants caution. Third, ethnic background and clinical practice variations may influence the efficacy of targeted therapy: the median rwPFS of Dab+Tram in the treatment-naïve FRONT-BRAF cohort was only 10.8 months, substantially lower than our PSM-adjusted 19.6 months. In addition, differences in co-mutational profiles, metastatic burden, drug accessibility, and the distribution of subsequent therapies may also confound cross-study comparisons. Our study is a single-centre retrospective analysis with a limited sample size after matching; therefore, the real-world evidence it provides is centred on the specific comparator of ICT and should be regarded as complementary to, rather than a refutation of, the FRONT-BRAF findings. Collectively, these studies underscore the urgent need for prospective trials incorporating molecular markers to guide individualised first-line therapy in BRAF-mutant NSCLC.

The PD-L1 subgroup analyses were exploratory and substantially limited by the high proportion of missing PD-L1 data (~66%) and the very small subgroup sizes. Although a statistically significant difference was observed in the PD-L1 1–49% subgroup, this finding is hypothesis-generating only and requires validation in larger, adequately powered cohorts with formal interaction testing (Supplementary Figure S2).

The trend linking treatment-emergent pyrexia to longer rwPFS warrants exploration. In this study, all pyrexia events occurred within the first 4 weeks of oral targeted therapy initiation (i.e., early-onset pyrexia). Patients with pyrexia had a median rwPFS of 32.5 months compared with 19.6 months in those without (difference 12.9 months, *p* = 0.895). Notably, the PSM-adjusted median rwPFS for the entire Dab+Tram cohort was 19.6 months, identical to the non-pyrexia subgroup. This suggests that the apparent benefit in pyrexial patients may partly reflect better baseline characteristics (e.g., better adherence or lower tumour burden) rather than a direct biological effect of fever. Preclinical studies have shown that BRAF/MEK inhibitors can remodel the tumour immune microenvironment by inducing immunogenic cell death [14], upregulating MHC-I expression [15], reducing MDSC infiltration [16] and releasing heat shock proteins [17,18]. Pyrexia, as a systemic inflammatory response, could theoretically synergistically amplify these immunomodulatory effects, but causality has not been established. Moreover, the fact that all pyrexia events occurred early after treatment initiation suggests that fever may reflect rapid attainment of therapeutic drug levels or individual susceptibility to the drug rather than sustained immune activation. Prospective studies with dynamic biomarkers [19] (e.g., IL-6, NLRP3 inflammasome) are needed to validate this association. The FRONT-BRAF study did not report on pyrexia-efficacy associations, but its safety analysis showed similar adverse event profiles for Dab+Tram regardless of treatment line [9], indirectly suggesting that pyrexia may not be a major determinant of treatment decisions.

Regarding later-line therapy, 14 patients in our study received second-line Dab+Tram, with a median rwPFS of 15.1 months; 9 patients received third-line or beyond, with a median rwPFS of 7.6 months. Although these data suggest that Dab+Tram retains antitumour activity after first-line non-targeted therapy failure, the sample sizes are extremely small (14 and 9, respectively) and the analysis is retrospective, with obvious selection bias (e.g., patients who could receive later-line Dab+Tram might have had better performance status and indolent disease biology). Therefore, interpretation of later-line efficacy must be extremely cautious; these results should be considered hypothesis-generating only, not definitive evidence. The BRF113928 study also reported that BRAF+MEK inhibitors used as second-line (after immunotherapy) had similar objective response rates and rwPFS to those used as first-line [5], but that study likewise lacked randomisation.

Based on the findings of the present study and emerging evidence from recent clinical trials, several promising future directions can be identified. First, the updated overall survival analysis from the phase II PHAROS study reported a median progression-free survival of 30.2 months in treatment-naive patients receiving encorafenib plus binimetinib, which is similar to the rwPFS of Dab+Tram observed in our cohort; the median overall survival was 47.6 months in treatment-naïve patients and 22.7 months in previously treated patients. These results highlight the potential of novel BRAF/MEK inhibitors in both first-line and second-line settings [20]. Second, the phase III OptiTROP-Lung05 interim analysis demonstrated that adding the anti-TROP2 antibody–drug conjugate (ADC) sacituzumab tirumotecan to pembrolizumab significantly improved rwPFS compared with pembrolizumab alone in patients with PD-L1-positive advanced NSCLC, representing the first ADC plus immune checkpoint inhibitor regimen to meet its primary endpoint in first-line NSCLC [21]. Collectively, these emerging data underscore the transformative potential of next-generation targeted therapies and ADC-based combinations, offering promising future strategies for both oncogene-driven and driver-negative NSCLC. Prospective head-to-head comparative studies are urgently needed to define the optimal first-line treatment sequence for BRAF V600-mutant NSCLC and to further evaluate the clinical value of BRAF/MEK inhibitors after failure of immunochemotherapy.

This study has several limitations. First, the single-centre retrospective design inevitably introduces selection bias and residual confounding. Although propensity score matching and multivariable Cox regression were applied to adjust for known covariates, unmeasured or incompletely recorded variables (e.g., smoking history, tumour mutational burden, and the distribution of PD-L1 expression in the ICT group) may still cause bias. The E-value analysis (E = 2.87 for HR = 1.74) suggests that it is unlikely that the observed effect can be entirely explained by unmeasured confounding, but residual confounding cannot be fully excluded. Second, smoking status is a well-established effect modifier of immunotherapy efficacy. In our study, smoking history could not be incorporated into the PSM model, limiting our ability to assess whether baseline smoking distribution was balanced between groups or whether ICT efficacy was partly attributable to smoking-related immunological advantages [22,23,24]. Third, the overall sample size was limited, with only 25 matched pairs after PSM, leading to insufficient statistical power for rwPFS and OS comparisons; in addition, subsequent crossover to targeted therapy upon progression may have further confounded between-group OS differences. Fourth, the PD-L1 subgroup analyses, later-line therapy analyses, and pyrexia-related analyses were all exploratory and constrained by very small subgroup sizes, thus serving only as hypothesis-generating. Finally, owing to the limited sample size, we could not perform stratified analyses by co-mutations (e.g., TP53, KRAS), which may influence prognosis and treatment response.

## 5. Conclusions

In this single-centre real-world cohort, first-line Dab+Tram showed a numerical rwPFS advantage over ICT and chemotherapy, but the difference was not statistically significant after PSM. Multivariable Cox analysis suggested a potential association with improved rwPFS, which requires validation in prospective studies. These findings provide complementary real-world evidence for first-line treatment selection in this rare molecular subgroup. Subsequent-line activity and pyrexia-related outcomes remain exploratory and require prospective validation.

## Figures and Tables

**Table 1:** Baseline characteristics of the study population.

Characteristics	Total (*n* = 142)	Dab+Tram (*n* = 47)	ICT (*n* = 30)	Che (*n* = 27)	Others (*n* = 15)	Dab+Tram-2L and LLT (*n* = 23)
Age, years (median, IQR)	65.0 (58.0–71.0)	68.0 (62.0–73.0)	62.6 ± 9.9	61.9 ± 9.1	64.7 ± 6.6	63.4 ± 8.3
Gender, n (%)						
Male	86 (60.6)	26 (55.3)	21 (70.0)	16 (59.3)	10 (66.7)	13 (56.5)
Female	56 (39.4)	21 (44.7)	9 (30.0)	11 (40.7)	5 (33.3)	10 (43.5)
Smoking history, n (%)						
Never	96 (67.6)	31 (66.0)	23 (76.7)	17 (63.0)	9 (60.0)	16 (69.6)
Current or former	46 (32.4)	16 (34.0)	7 (23.3)	10 (37.0)	6 (40.0)	7 (30.4)
BMI, kg/m^2^ (median, IQR)	23.4 (20.8–23.4)	23.5 ± 3.0	23.2 ± 2.9	22.5 ± 3.7	22.2 ± 3.5	23.1 ± 3.3
NA, n	10	4	1	2	1	2
Pathology, n (%)						
Adenocarcinoma	138 (97.2)	45 (95.7)	28 (93.3)	27 (100.0)	15 (100.0)	23 (100.0)
Non-adenocarcinoma	4 (2.8)	2 (4.3)	2 (6.7)	0 (0.0)	0 (0.0)	0 (0.0)
Stage at initial diagnosis, n (%)						
Stage III	19 (13.4)	5 (10.5)	4 (13.3)	4 (14.8)	2 (13.3)	4 (17.3)
Stage IV	123 (86.6)	42 (89.5)	26 (86.7)	23 (85.2)	13 (86.7)	19 (82.7)
PD-L1 expression, n (%)						
<1%	8 (16.7)	1 (5.9)	2 (33.3)	2 (13.4)	1 (20.0)	2 (40.0)
1–49%	19 (39.6)	7 (41.2)	7 (46.6)	3 (50.0)	1 (20.0)	1 (20.0)
≥50%	21 (44.7)	9 (52.9)	6 (40.0)	1 (16.7)	2 (40.0)	3 (60.0)
NA, n	94	30	15	21	10	18
Pleura metastasis, n (%)						
No	86 (60.6)	30 (63.9)	15 (50.0)	17 (63.0)	10 (66.7)	14 (60.9)
Yes	56 (39.4)	17 (36.1)	15 (50.0)	10 (37.0)	5 (33.3)	9 (39.1)
Bone metastasis, n (%)						
No	107 (75.4)	36 (76.6)	20 (66.7)	21 (77.7)	12 (80.0)	18 (79.3)
Yes	35 (24.6)	11 (23.4)	10 (33.3)	6 (22.3)	3 (20.0)	5 (21.7)
Brain metastasis, n (%)						
No	132 (93.0)	44 (93.7)	30 (100.0)	24 (88.9)	13 (86.7)	21 (91.3)
Yes	10 (7.0)	3 (6.3)	0 (0.0)	3 (11.1)	2 (13.3)	2 (8.7)
Adrenal gland metastasis, n (%)						
No	132 (93.0)	45 (95.8)	26 (86.7)	26 (96.3)	15 (100.0)	20 (87.0)
Yes	10 (7.0)	2 (4.2)	4 (13.3)	1 (3.7)	0 (0.0)	3 (13.0)
BRAF mutation type, n (%)						
V600E	109 (94.0)	43 (100.0)	20 (90.9)	16 (84.2)	12 (85.7)	18 (100.0)
Non-V600E	7 (6.0)	0 (0.0)	2 (9.1)	3 (15.8)	2 (14.3)	0 (0.0)
NA, n	26	4	8	8	1	5
Any co-mutation, n (%)						
No	105 (85.4)	38 (88.4)	19 (76.0)	15 (71.4)	14 (93.3)	19 (100.0)
Yes	18 (14.6)	5 (11.6)	6 (24.0)	6 (28.6)	1 (6.7)	0 (0.0)
NA, n	19	4	5	6	0	4

Dab+Tram—Dabrafenib plus trametinib; ICT—Immunochemotherapy; Che—Chemotherapy; 2L—Second-line treatment; Others—Immunotherapy-based regimens (immunotherapy monotherapy and immunotherapy plus anti-angiogenic therapy, etc.); BMI—Body Mass Index; NA—Not available; LLT—later-line therapy.

**Table 2:** Basic clinicopathological characteristics before and after propensity score matching.

	Total Cohort				PSM Cohort			
Characteristic	Total (77)	Dab+Tram (47)	ICT(30)	SMD	Total (50)	D+T (25)	ICT (25)	SMD
Age				0.42				<0.001
<65	34 (44.2%)	17 (36.2%)	17 (56.7)		26 (52.0)	13 (52.0)	13 (52.0)	
≥65	43 (55.8%)	30 (63.8%)	13 (43.3)		24 (48.0)	12 (48.0)	12 (48.0)	
Gender				0.307				0.091
Male	47 (61.0%)	26 (55.3%)	21 (70.0)		37 (74.0)	19 (76.0)	18 (72.0)	
Female	30 (39.0%)	21 (44.7%)	9 (30.0)		13 (26.0)	6 (24.0)	7 (28.0)	
ECOG				0.411				0.134
0	18 (23.4%)	14 (29.8%)	4 (13.3)		5 (10.0)	3 (12.0)	2 (8.0)	
1	54 (70.1%)	30 (63.8%)	24 (80.0)		41 (82.0)	20 (80.0)	21 (84.0)	
2	5 (6.5%)	3 (6.4%)	2 (6.7)		4 (8.0)	2 (8.0)	2 (8.0)	
cstage				0.083				0.169
III	9 (11.7%)	5 (10.6%)	4 (13.3)		3 (6.0)	2 (8.0)	1 (4.0)	
IV	68 (88.3%)	42 (89.4%)	26 (86.7)		47 (94.0)	23 (92.0)	24 (96.0)	

ECOG—Eastern Cooperative Oncology Group; Dab+Tram—Dabrafenib plus trametinib.

**Table 3:** Univariate and multivariable Cox regression for rwPFS and OS.

Variable	OS				rwPFS			
	Univariate Analysis		Multivariate Analysis		Univariate Analysis		Multivariate Analysis	
	HR (95% CI)	*p*-Value	HR (95% CI)	*p*-Value	HR (95% CI)	*p*-Value	HR (95% CI)	*p*-Value
Age	0.997 (0.962–1.037)	0.863	0.998 (0.966–1.037)	0.930	0.986 (0.967–1.006)	0.160	0.992 (0.971–1.015)	0.500
Gender	0.462 (0.162–1.108)	0.086	0.716 (0.216–2.147)	0.559	1.294 (0.792–2.076)	0.298	1.638 (0.917–2.923)	0.095
ECOG	4.872 (1.279–13.690)	0.024	3.650 (0.829–13.053)	0.083	2.380 (0.873–5.251)	0.085	1.679 (0.567–4.190)	0.324
Smoking status	2.173 (0.991–4.729)	0.053	0.592 (0.237–1.430)	0.244	0.813 (0.483–1.328)	0.416	1.120 (0.623–2.039)	0.707
Clinical stage	1.259 (0.453–4.810)	0.686	0.724 (0.212–3.073)	0.633	1.651 (0.822–3.858)	0.169	1.085 (0.500–2.670)	0.845
Extrathoracic metastasis	2.665 (1.227–5.785)	0.014	2.246 (0.839–6.142)	0.119	1.729 (1.064–2.784)	0.027	2.029 (1.152–3.540)	0.015
Treatment a	0.580 (0.191–1.557)	0.286	0.544 (0.161–1.621)	0.281	2.533 (1.363–4.764)	0.003	2.500 (1.322–4.780)	0.005
Treatment b	1.134 (0.427–2.841)	0.792	1.591 (0.563–4.294)	0.369	5.074 (2.684–9.734)	<0.001	5.534 (2.900–10.706)	<0.001
Treatment c	0.921 (0.233–2.810)	0.893	0.799 (0.196–2.536)	0.719	2.181 (0.981–4.603)	0.056	2.652 (1.170–5.724)	0.021

Abbreviations: CI, confidence interval; ECOG, Eastern Cooperative Oncology Group; HR, hazard ratio. Smoking status: Current or former vs. never. Clinical stage: IV vs. III. a: ICT vs. D+T. b: Chemotherapy vs. D+T. c: Others vs. D+T.

## Data Availability

The datasets generated and/or analysed during the current study are not publicly available due to institutional privacy policies and restrictions related to patient confidentiality. However, they are available from the corresponding author upon reasonable request and with permission from Shanghai Pulmonary Hospital, Tongji University.

## Supplementary Materials

The following supporting information can be downloaded at https://www.mdpi.com/article/10.3390/jcm15176749/s1, Supplementary Figure S1. rwPFS in patients with locally advanced (stage III) BRAF V600-mutant NSCLC receiving first-line Dab+Tram versus chemotherapy alone. Supplementary Figure S2. Exploratory subgroup analysis of rwPFS and OS according to PD-L1 expression in patients receiving first-line Dab+Tram versus ICT. Supplementary Figure S3. rwPFS of subsequent-line Dab+Tram after first-line non-targeted treatment failure in BRAF V600-mutant NSCLC.

## Abbreviations

The following abbreviations are used in this manuscript:

NSCLC	Non-small-cell lung cancer
ICT	Immunochemotherapy
ADC	Antibody–drug conjugate
Dab+Tram	Dabrafenib plus trametinib
rwPFS	Real-world progression-free survival
OS	Overall survival
HR	Hazard ratio
CI	Confidence interval
PSM	Propensity score matching
ECOG	Eastern Cooperative Oncology Group
NGS	Next-generation sequencing
PCR	Polymerase chain reaction
PD-L1	Programmed death-ligand 1
