# Supplementary material for: Real-World Comparative Effectiveness of First-Line Dabrafenib Plus Trametinib Versus Immunochemotherapy in Patients with Advanced BRAF V600-Mutant Non-Small-Cell Lung Cancer: A Retrospective Cohort Study
Source: J Clin Med. 2026 Aug 31;15(17):6749. doi: 10.3390/jcm15176749 (PMC13566382; doi:10.3390/jcm15176749)
Supplement: Supplementary file 1 [file jcm-15-06749-s001.zip › jcm-4467161-supplementary.pdf]

Supplementary Materials

Supplementary Figure S1. rwPFS in patients with locally advanced (stage III) BRAF V600-mutant NSCLC receiving first-line Dab+Tram versus chemotherapy alone. The Kaplan-Meier curves show rwPFS for the stage III subgroup (n=19). Dab+Tram achieved a median rwPFS of 34.7 months (95% CI: 28.9-not reached) compared with 3.4 months (95% CI: 1.2–6.8) for chemotherapy (log-rank $P < 0.001$). The number of patients at risk is shown below the curves.

Supplementary Figure S2. Exploratory subgroup analysis of PFS and OS according to PD-L1 expression in patients receiving first-line Dab+Tram versus ICT. Kaplan-Meier estimates for PD-L1 1-49% subgroup (rwPFS, $P = 0.034$; OS, $P = 0.34$) and PD-L1 ≥50% subgroup (both not significant). Number of patients at risk is shown below the curves.

Supplementary Figure S3. rwPFS of subsequent-line Dab+Tram after first-line non-targeted treatment failure in BRAF V600-mutant NSCLC. Median PFS was 15.1 months in 14 patients receiving second-line Dab+Tram and 7.6 months in 9 patients receiving third-line or beyond. The number of patients at risk is shown below the curves.

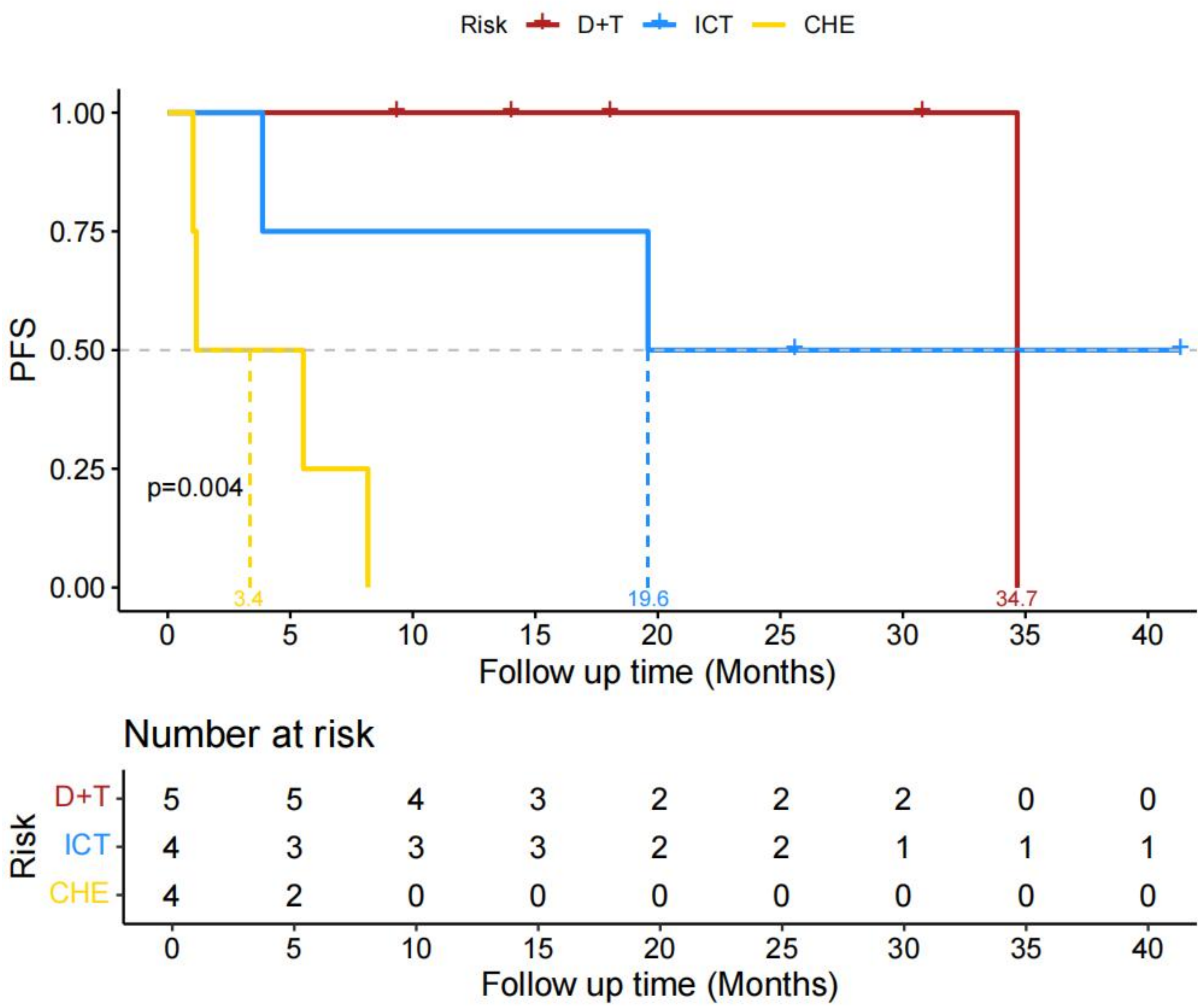

Risk
D+T
ICT
CHE
PFS
1.00
0.75
0.50
0.25
0.00
p=0.004
3.4
19.6
34.7
0
5
10
15
20
25
30
35
40
Follow up time (Months)
Number at risk
Risk
D+T 5 5 4 3 2 2 2 0 0
ICT 4 3 3 3 2 2 1 1 1
CHE 4 2 0 0 0 0 0 0 0
Follow up time (Months)

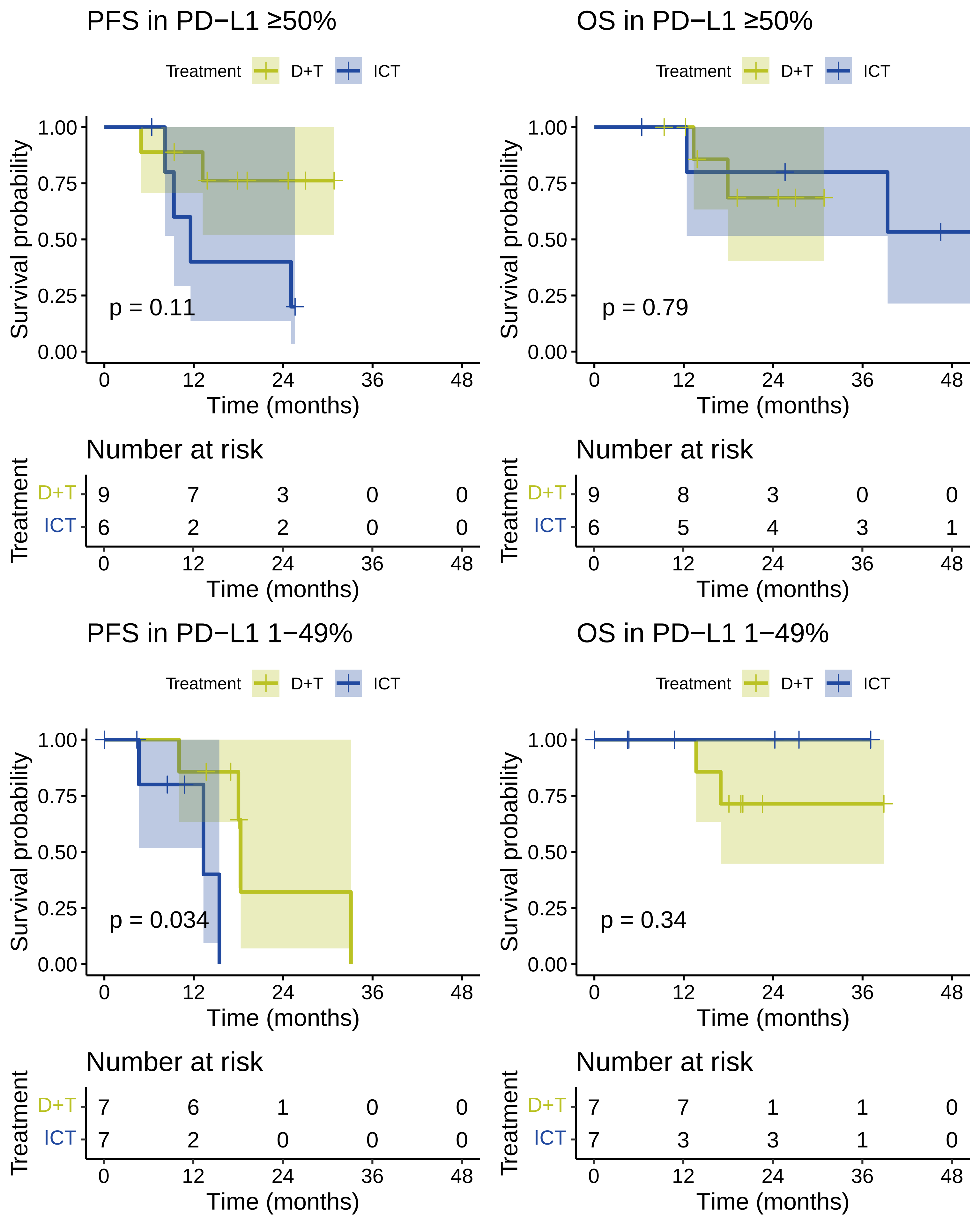
PFS in PD−L1 ≥50%
Treatment D+T ICT
Survival probability
1.00 0.75 0.50 0.25 0.00
p = 0.11
0 12 24 36 48
Time (months)
Number at risk
Treatment
D+T 9 7 3 0 0
ICT 6 2 2 0 0
OS in PD−L1 ≥50%
p = 0.79
Number at risk
D+T 9 8 3 0 0
ICT 6 5 4 3 1
PFS in PD−L1 1−49%
p = 0.034
Number at risk
D+T 7 6 1 0 0
ICT 7 2 0 0 0
OS in PD−L1 1−49%
p = 0.34
Number at risk
D+T 7 7 1 1 0
ICT 7 3 3 1 0

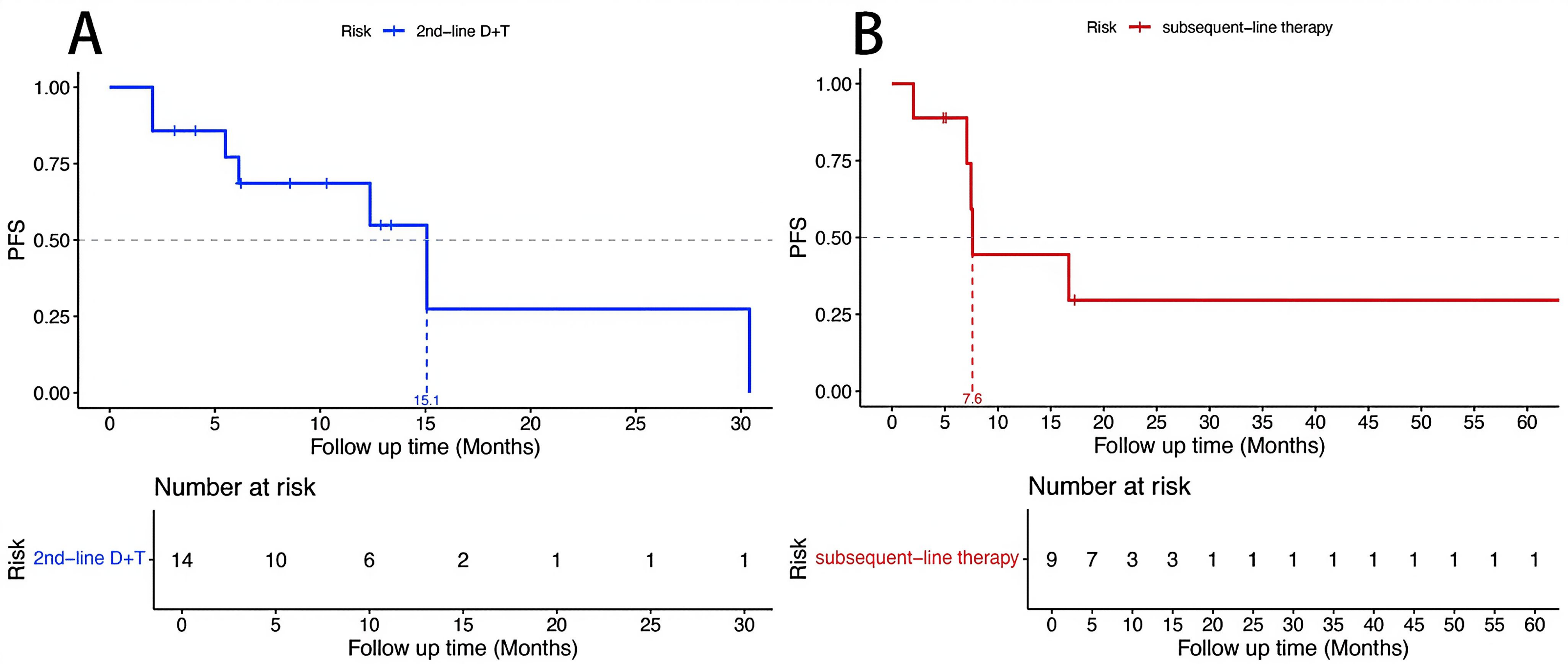

A
Risk 2nd–line D+T
PFS
1.00
0.75
0.50
0.25
0.00
15.1
0 5 10 15 20 25 30
Follow up time (Months)
Number at risk
Risk
2nd–line D+T 14 10 6 2 1 1 1
0 5 10 15 20 25 30
Follow up time (Months)
B
Risk subsequent–line therapy
PFS
1.00
0.75
0.50
0.25
0.00
7.6
0 5 10 15 20 25 30 35 40 45 50 55 60
Follow up time (Months)
Number at risk
Risk
subsequent–line therapy 9 7 3 3 1 1 1 1 1 1 1 1 1
0 5 10 15 20 25 30 35 40 45 50 55 60
Follow up time (Months)
